# Vaginal and rectal microbiome changes following administration of a multi-species antenatal probiotic: A randomized control trial

**DOI:** 10.1080/29933935.2024.2334311

**Published:** 2024-04-19

**Authors:** Emily Malloy, Ashley E. Kates, Jonah Dixon, Colleen Riley, Nasia Safdar, Lisa Hanson

**Affiliations:** a Aurora UW Medical Group Midwifery & Wellness, Advocate Aurora Healthcare Milwaukee, Milwaukee, USA; b College of Nursing, Marquette University, Milwaukee, USA; c Department of Medicine, Division of Infectious Disease, University of Wisconsin-Madison, Madison, USA; d Department of Medicine, William S. Middleton Memorial Veterans Hospital, Madison, USA

**Keywords:** Pregnancy, probiotics, group B Streptococcus, vaginal microbiome, racial disparities

## Abstract

The gut and vaginal microbiome undergo changes during pregnancy which may be protective or harmful to the birthing person. Probiotics have been found to cause protective changes to the gut and vaginal microbiomes, with the potential to improve perinatal outcomes. This randomized control trial (NCT03696953) compares the vaginal and rectal microbiomes before and after an antenatal probiotic or placebo intervention, with a diverse group of pregnant people and a special focus on racial disparities. The vaginal and rectal microbiomes reveal non-significant increased *Lactobacillus* in the probiotics group, with a greater increase in participants who identified as Black. Potential implications and future studies are discussed.

## Introduction (& Lit Review)

In pregnant people, the microbiome of the gut and vagina change dramatically between the first and third trimesters due to complex hormonal and physiologic interactions.^1–6^ Changes include increased β-diversity (differences between pregnant people), an overall increase in proteobacteria and actinobacteria, and a lower number of species occurs, also known as reduced richness or a-diversity.^7^ During the course of normal pregnancy, the vaginal microbiome composition changes substantially; a-diversity decreases, stability of microbiota increases, and the vagina is enriched with *Lactobacillus* species.^8–11^ Vaginal secretions are increased and pH becomes more acidic. An acidic vaginal pH promotes balance of commensal organisms to prevent pathogen adherence.^12^ The presence of *Bifidobacterium* is associated with a healthy and diverse GI microbiota.^13^ By the third trimester, the vaginal microbiota resembles that of the nonpregnant state.^8^ Microbiome diversity may increase at the time of labor.^14^ These physiologic changes in the microbiome likely contribute to healthy pregnancy outcomes.^15^

A variety of factors contribute to the composition of the vaginal microbiome including: genetics, diet, sexual activity, vaginal hygiene practices, antibiotic use, substance use, hormonal contraception, stress, race, ethnicity, education, employment in healthcare, and neighborhood.^16,17^ *Lactobacillus* species that colonize the vagina produce acids and other substances that are protective against harmful microbes. Colonization with *Lactobacillus* is considered a marker of a healthy vaginal microbiome, although not all species are equally protective.^16^ Variations in the vaginal microbiome have been identified between different racial and ethnic groups, although *Lactobacillus* species appear to be the most common.^18,19^

Group B *Streptococcus* (GBS) is a gram-positive coccus that resides in the GI and urogenital tracts and has been a part of the human microbiome since the 1970s. GBS vaginal/rectal colonization in pregnant people is reported in the range of 20–30%, with highest rates reported for those of African descent.^20,21^ Vaginal/rectal GBS colonization in late pregnancy is associated with vertical transfer to neonates. GBS carriage is also associated with disease in nonpregnant individuals and is present at similar rates as in pregnancy.^22^ Early onset GBS neonatal disease is associated with severe neonatal morbidity and mortality, and risk factors include Black race, and young maternal age.^23^ A study of pregnant people who carry GBS was compared to GBS negative participants and found the vaginal microbiome of GBS carriers was more diverse and less homogenous. Both alpha-diversity and beta-diversity were significantly different, with significantly more phylum Firmicutes and *Lactobacillus* species in the GBS negative participants.^24,25^

Probiotic bacterial species like those contained in over the counter probiotic supplements colonize mucosal surfaces like the vagina and GI tract where they secrete substances such as acids, vitamins, and bacteriocins that prevent pathogen adherence.^26,27^ The presence of *Lactobacillus* and other lactic acid producing bacteria^28^ is considered a marker for vaginal health^29^ and is associated with a reduction in vaginal pathogens like GBS.^30–33^ Oral probiotic interventions can modify the vaginal microbiota within 2–10 days.^33–36^ Several randomized controlled trials have been conducted to determine the efficacy of probiotics to reduce GBS colonization.^32,37–40^

Here, we present the results of a clinical trial assessing the impact of antenatal probiotics on the vaginal and gut microbiomes.^32^ One specific aim of the trial was to determine the efficacy of oral probiotics taken once daily during pregnancy for a total of ±10 weeks beginning at 28 (±2) weeks gestation compared with identical placebo to improve the maternal vaginal and rectal microbiome. Participation was limited to those with low-risk pregnancies. We hypothesized that participants in the intervention (probiotics) group will have higher *Lactobacillus* colony counts at 35–37 weeks gestation compared to their baseline counts at 28 weeks and to those in the placebo group.

## Results

Vaginal and rectal microbiome and GBS swabs were collected on all participants at 28 weeks (±2 weeks) gestation who agreed to voluntarily enroll in the study, prior to initiation of the probiotic or identical placebo capsule (T1). The vaginal and rectal microbiome and GBS swabs were repeated at 36 weeks (±2 weeks) gestation after daily ingestion of the probiotic or identical placebo capsule (T2) for ±10 weeks. A total of 83 participants completed the study, *N* = 39 in the probiotic group and *N* = 44 in the placebo group. Adherence to the probiotic intervention was 51% and to the placebo was 60% and the probiotic intervention reduced antenatal GBS colonization by 20%, although the study was underpowered to find a statistically significant difference.^32^

**Figure 1.**
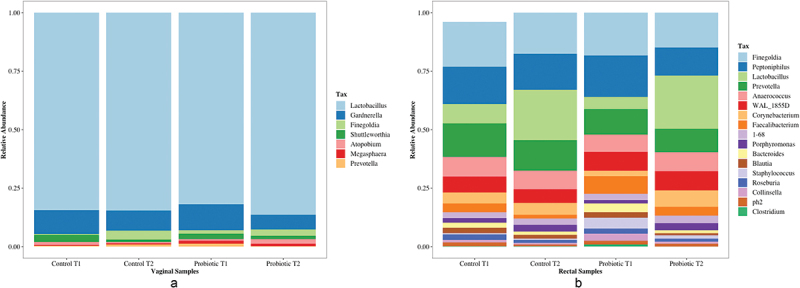
Relative abundance bar plots of the top genera aggregated by group and timepoint for the (a) vaginal and (b) rectal samples. ASVs with less than 100 reads present in the dataset were removed for visualization.

The vaginal microbiome was dominated by *Lactobacillus* in both the control and probiotic groups as shown in Fig. 1a. There was little change in *Lactobacillus* abundance between T1 and T2, although there was an increase in the probiotic group at T2. Both groups had an increase in *Finegoldia* and decreases in *Gardnerella* and *Shuttleworthia*, although the decrease in *Gardnerella* is larger in the probiotics group at T2. While *Prevotella* was in low abundance in both groups across both timepoints, there was a slight increase in the control group at T2 compared to a decrease in the probiotics group. In the probiotics group, *Clostridium* was eliminated. In the rectal swabs, there was greater diversity with 17 genera present across the top 100 ASVs. While there were very minor changes to the abundances T1 and T2, there was an increase in *Lactobacillus* in both groups at T2 (Fig. 1b). Vaginal samples ranged from 16 reads to 103,473. Rectal samples ranged from 2 to 95,014 reads. Histograms of the read density are included. During the DNA extraction and quantitation process it was noted that several samples were of very low biomass. As some of these samples were collected by clinicians and others were self-collected, we suspect there were issues with adequate swabbing resulting in falsely low abundance.

**Figure 2.**
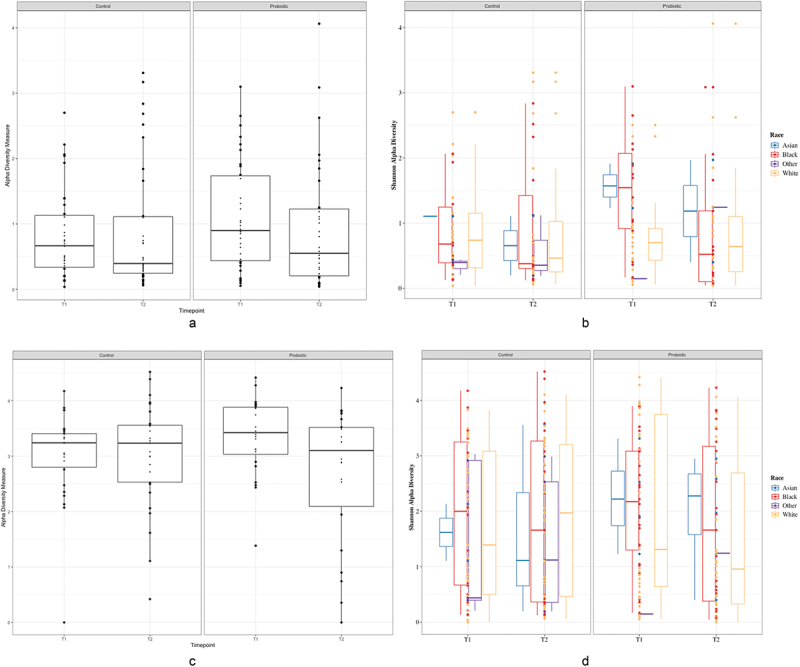
Shannon alpha diversity box and whisker plots for all samples. (a) vaginal alpha diversity by group and time point. (b) vaginal alpha diversity by group and timepoint showing differences by race. (c) rectal alpha diversity by group and time point. (d) rectal alpha diversity by group and timepoint showing differences by race.

Vaginal Shannon diversity (Figs. 2a,b) ranged from 0.037–2.70 at T1 and 0.063–3.31 at T2 in the control group and 0.056–3.098 at T1 and 0.042–4.062 at T2 in the probiotic group. There were no significant differences between timepoints for either group, however there was a near significant decrease in alpha diversity between T1 and T2 for the probiotic group (*p* = 0.086). Vaginal and rectal alpha p-values are in Table 1. For the rectal samples, Shannon diversity ranged from 0.0–4.17 at T1 and 0.42–4.52 at T2 in the control samples and 1.38–4.42 at T1 and 0–4.23 at T2. In both the probiotic and control groups, there were no changes in rectal site alpha diversity between time points. In the rectal samples, the greatest differences in alpha diversity were observed when considering race (Fig. 1b). There was a nonsignificant decrease in Shannon diversity between timepoints in the probiotic group for those identifying as Black or Asian compared to those identifying as White (*p* = 0.395).

**Table 1.** Vaginal and Rectal alpha* p-values for control v probiotic group.

	P-value
**Vaginal Groups**	
Control T1 vs. control T2	0.999
Control T1 vs probiotic T1	0.358
Control T2 vs probiotic T2	0.826
Probiotic T1 vs probiotic T2	0.0857
**Rectal Groups**	
Control T1 vs. control T2	0.936
Control T1 vs probiotic T1	0.992
Control T2 vs probiotic T2	0.296
Probiotic T1 vs probiotic T2	0.822

**Note**. *Alpha diversity p-values were calculated using ANOVA with the Tukey HSD test).

**Figure 3.**
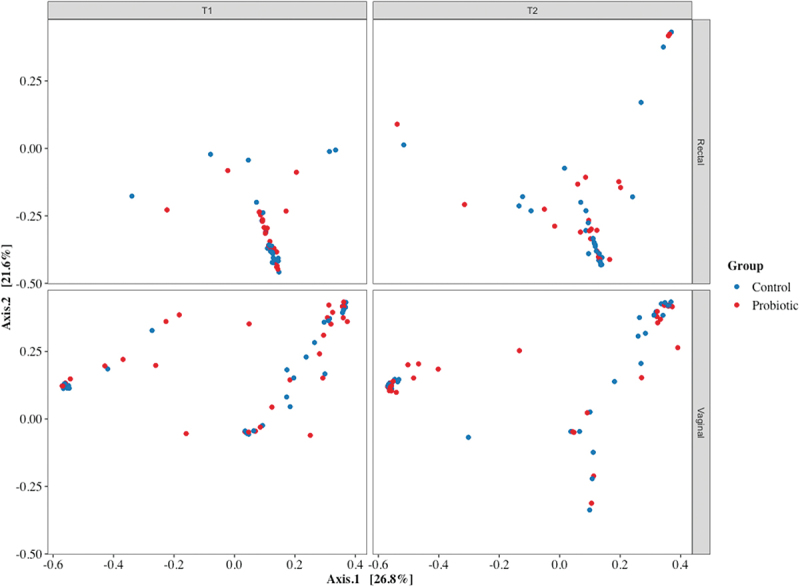
Principal coordinates analysis (PCoA) of the Bray-Curtis dissimilarity index with the Benjamini-Hochberg correction applied. The relative contribution (eigenvalue) of each axis to the total inertia in the data is indicated in the percent values in each axis title. Distances between points indicates the difference in ASV composition in samples. (a) by sample site (vaginal vs rectal) and (b) group and timepoint.

To assess beta diversity, the Bray-Curtis dissimilarity was calculated and visualized by PCoA (Fig. 3). For the vaginal samples, there were no differences by group (*p* = 0.604) or timepoint (0.418). The same was true for the rectal samples (group p-value = 0.581 and timepoints p-value = 0.328). PERMANOVA assumes homoscedasticity and the data did not show significantly different dispersions for either the vaginal (*p* = 0.329) or rectal (*p* = .371) samples.

**Figure 4.**
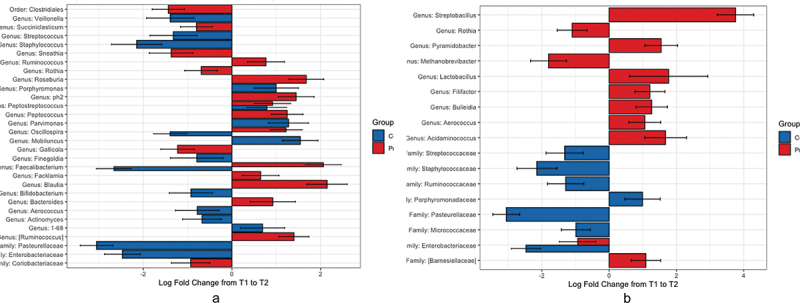
Taxa differentially abundant between T1 and T2 represented by the effect size (log fold change) and their 95% confidence interval bars from ANCOM-BC for the a) vaginal and b) rectal samples. The log fold change values represent the difference in abundance in the taxa between timepoints 1 and 2. Not all taxa were differentially abundant in both groups. All effect size p-values have the Benjamini-Hochberg correction applied. Control samples are colored in blue and probiotic group samples are colored in red.

Differential abundance testing identified 33 (17 in the probiotic group, 16 in the control group) differential abundant taxa in the vaginal samples (Fig. 4a) and 18 (11 in the probiotic group, 7 in the control group) in the rectal samples (Fig. 4b).

## Discussion

In this study, there was near a near-significant difference (p = 0.086) in vaginal microbiome diversity, possibly due to the increase in *Lactobacillus*, with the largest difference seen in Black probiotics group participants. As reported previously, adherence to the daily study intervention 51% in the probiotics group and 60% in the control group, (Cohen’s d = 0.230; 95% CI 0.203 to 0.661; p = .3)^32^ but despite low adherence there was lower alpha diversity in the probiotics group, while in the control group there was no change. *Lactobacillus* is the dominant vaginal probiotic bacterial species in 80–90% of Asian and White women in the USA. In contrast, the vaginal microbiomes of Hispanic and Black women are composed of 60% *Lactobacillus* species, along with other lactic acid producing genera such as *Megasphaera*, *Streptococcus*, and *Atopobium*.^28^ Vaginal pH has been found to be higher in Black (pH 4.7 ± 1.04) and Hispanic women (pH 5.0 ± 0.59), which may contribute to increased colonization with pathogenic bacteria, including GBS. Pregnant people of African descent have a higher prevalence of GBS colonization compared to Whites in the USA.^41^ This health disparity extends globally with the highest rates of GBS worldwide in the Caribbean and in several African countries.^21^ Differences in the vaginal microbiome may have a role in rates of GBS colonization, although these differences may also be a physiologic or environmental variation or play a not yet understood protective role. In this study, we observed that the microbiomes of Black and White women became more similar to each other in the probiotics group. Clinical trial demographic data are in Table 2. Although the clinical trial did not have a statistically significant primary outcome, there was a reduction in GBS colonization by 20% and oral probiotic interventions may represent a means to manipulate the vaginal and gut microbiome to a more protective state during pregnancy especially for Black women who experience disproportionately poorer perinatal outcomes in the USA due to social determinants of health.^42,43^

**Table 2.** Demographic and outcomes of study participants.

Variable	Probiotics *(N=38)*	Placebo *(N=44)*	*p* Value
Age (years), Mean (SD)	28.7 (5.4)	28.5 (5.4)	0.8755
Race			.3934
Asian	2	2	
Black	17	16	
White	19	23	
Did not answer	0	2	
More than one race	0	1	
Ethnicity			0.5069
Hispanic	2	4	
Non-Hispanic	36	40	
*Parity, N, %*			0.8504
Nulliparous	16 (4.1)	19 (44.2)	
Multiparous (Missing =1)	22 (57.9)	24 (55.8)	
*Mode of Birth*			0.6277
Vaginal	31(81.6)	33 (78.6)	
Cesarean	7 18.4)	8 (19.1)	
Vacuum Missing =2	0 (0.0)	1 (2.4)	
*Apgar Scores, Mean, SD*			
1 min	7.6 (1.3)	7.6 (1.1)	0.9666
5 min	8.8 (0.5)	8.8 (0.6)	0.8123
*Neonatal Resuscitation, N,%*			0.4060
Yes	7 (18.4)	11 (26.2)	
No *Missing =2*	31(81.8)	31 (73.8)	
*Neonate, Mean, SD*			
Birth weight	3376.4 (483.0)	3366.7 (448.0)	0.9261
Length	51.6 (2.8)	52.2 (2.8)	0.3060
Head Circumference	34.2 (1.5)	34.0 (1.8)	0.6072
Average AP-GI-SA score *Mean, SD*			
Baseline 28 weeks	14.7 (3.2)	16.2 (4.8)	0.996
36 weeks	13.58 (2.77)	15.64 (3.96)	0.0081
Post Birth (missing data)	13.5 (4.13)	13.56 (2.66)	0.9509

Hanson L, VandeVusse L, Forgie M, et al. A randomized controlled trial of an oral probiotic to reduce antepartum group B Streptococcus colonization and gastrointestinal symptoms. *Am J Obstet Gynecol MFM*. Jan 2023;5(1):100748. https://doi.org/10.1016/j.ajogmf.2022.100748.^32^

There were no significant differences by race in either the vaginal or rectal samples, likely due to small numbers and low adherence to study capsules (probiotic and placebo).

However, there was an observed, nonsignificant decrease in Shannon alpha diversity for Black participants in the probiotics group. Increased *Lactobacillus* was more apparent in the rectal swabs in the probiotics group, although it was also present in the control group. The less abundant groups had more changes and differences between groups and are more apparent in less abundant taxa. *Clostridium* disappeared entirely in the probiotics group, which is potentially beneficial as this is a genus with several pathogens, although unrelated to the pathogen *C. difficle—*part of the *Clostridioides* genus. There were no differences in beta diversity.

The role of probiotics to modify the gut and vaginal microbiome of pregnant people continues to evolve. Authors of two recent systematic reviews/meta-analyzes found reduced rates of vaginal/rectal GBS colonization among participants who took probiotics.^33,44^ A small RCT of pregnant people with vaginal dysbiosis were treated with vaginal probiotics (*Lactobacillus casei rhamnosus*) had increased gestational age and neonatal birth weights compared to controls.^45^ Probiotics may have a role in decreasing inflammation during pregnancy and therefore decreasing preterm birth, although not all species of *Lactobacillus* are useful for this and a combination of multiple strains of *Lactobacillus* and *Bifidobacterium* appear to be more useful^46^ along with protection against several adverse pregnancy outcomes.^47,48^ A small longitudinal study found that *Lactobacillus* remains dominant during the third trimester and at birth.^49^ The role of probiotics in decreasing rates of preterm birth is unclear.^50,51^

Bacterial vaginosis (BV) has long been associated with adverse pregnancy outcomes, although the exact mechanism of action is poorly understood. *Gardnerella vaginalis* (GV) is an anaerobic bacterial species that causes vaginal dysbiosis and is associated with poor pregnancy outcomes.^52^ Although both the control and probiotics groups experienced a non-significant decrease in *Gardnerella* at T2, the decrease is larger in the probiotics group with a concurrent increase in *Lactobacillus*. This microbiome change may be protective against some adverse events of pregnancy, particularly in the context of known racial disparities between Black and White birthing people in the US, as prior studies have found a higher vaginal pH in Black and Hispanic people^28^ and deserve further study.

In our previously published study^32^ the 10-item Antepartum Gastrointestinal Symptom Inventory (AP-GI-SA)^53^ was administered to study participants. After ±10 weeks of the intervention beginning at 28 weeks, probiotic group participants had a significant reduction in GI symptoms of pregnancy compared (13.7 ± 2.9) to controls (15.6 ± 3.9) at 36 weeks gestation (Cohen’s d-0.528; 95% CI, 0.087–1.088; *p* = 0.0081). This may have been related to the increase in the abundance of *Lactobacillus* in the rectal swabs in the probiotics group, excluding other potentially symptom causing microbes. GI symptoms of pregnancy represent an understudied area in microbiome research and studies analyzing the microbiome may offer insight into improving symptoms for pregnant people.

## Possible implications/conclusions

Pregnant participants tolerated the probiotic intervention with no adverse events, as monitored by the data safety monitoring board and clinical research coordinator. Adherence to the probiotic and placebo capsules was low (average 55% of capsules taken) and not significantly different between groups. Microbiome swabs were obtained at baseline (28 weeks) and compared to those taken at 36 weeks gestation as a part of a randomized placebo-controlled trial. Participants in the probiotic group experienced a nonsignificant increase in the abundance of *Lactobacillus* which suggests the mechanism of action of the probiotic intervention. The microbiome of Black participants was enhanced with increased *Lactobacillus* by the probiotic over the placebo. Probiotic interventions containing *Lactobacilli* may present opportunities for future research toward manipulating the gut and vaginal microbiomes to improve perinatal outcomes and reduce GBS colonization. Larger studies of this intervention are recommended, including those with microbiome analysis. Prebiotic studies including dietary fiber and recall represent an additional area for study.

## Patients and methods/materials and methods

This study was part of an NIH-funded, double-blind, randomized, placebo-controlled trial, “The Efficacy of Probiotics to Reduce Antepartum GBS” (R21HD095320, clinical.trials.gov Identifier: NCT03696953), which has been published.^32^ The study received ethics approval from three institutional review boards (IRBs), including the hospital system of the study setting, the university of the laboratory site co-investigators, and the university of principal and co-investigators. The aim of the parent study was to demonstrate the efficacy of an oral probiotic intervention to reduce GBS colonization at 36 weeks gestation, and therefore, lessen the need for intrapartum antibiotic prophylaxis. After informed consent, participants were randomly assigned to either Florajen3 probiotic capsules (*Lactobacillus acidophilus*, *Bifidobacterium lactis*, and *Bifidobacterium longum*) in a microcrystalline cellulose carrier (MCC) or a placebo (MCC) that was identical in taste and appearance. The study capsules were initiated at 28 ± week gestation and taken once daily until the time of labor and giving birth. The aim of this study was to identify microbiota communities in the vagina and rectum of pregnant participants. Because probiotic bacteria colonize mucosal surfaces within the vagina and gastrointestinal tract, we hypothesized that the probiotics would promote an increase in beneficial microbes and reduce pathogen adherence.

### DNA extraction and sequencing

The detailed descriptions of DNA extraction methods have been previously published.^54^ Briefly, total genomic DNA was extracted using a bead-beating protocol with additional enzymatic lysis containing mutanolysin, lysostaphin, and lysozyme to assist in lysing gram-positive cell walls. For the rectal swabs, an additional phenol:chloroform:isoamyl alcohol was added after the mechanical lysis for additional DNA clean-up. 500 µL phenol:chloroform:isoamyl alcohol was added to the sample along with enough TSE buffer to bring the final volume to 1.2 mL. Tubes were then centrifuged for 10 minutes at 4°C at 16,000 × g. The aqueous layer was then transferred to a clean 2 mL tube and the phenol:chloroform:isoamyl alcohol wash step was repeated on more time. Samples were purified using the NucleoSpin Gel and PCR cleanup kit according to the manufacturer’s directions (Macherey-Nagel, Germany) and stored at −80°C. DNA was quantified using the Qubit dsDNA kit (Invitrogen, Waltham, MA, USA) on the Biotek Synergy HTX (Biotek Instruments, Winooski, VT, USA). Samples were then sequenced using 16S rRNA sequencing of the V4 region on the Illumina MiSeq at the University of Wisconsin Biotechnology Center. Purified DNA was normalized to 5 ng/μL, amplified using barcoded primers for the 16S V4 region, and sequenced using 2 × 250 paired end reads.

### Bioinformatic and statistical analyses

Sequence quality was assessed using FASTQC.^55^ Sequences with low average base quality scores, short reads less than 150 bases, reads with uncalled bases, and sequences that could not assemble into paired reads were removed from the dataset. Raw sequences were processed into amplicon sequencing variants (ASVs) using QIIME2 following the “Moving Pictures” protocol.^56^ DADA2^57^ was used for the quality control steps. Taxonomy was assigned using the GreenGenes database to the genus level whenever possible. All statistical analyzes were conducted in R using version 4.3.1.^58^ Alpha diversity was assessed using the Shannon and Inverse Simpson diversity indices. ANOVA was used to determine significant differences in alpha diversity, and the Tukey’s honest significance test was used to correct for multiple comparisons. The Bray-Curtis dissimilarity matrices were used to assess beta diversity and visualized using PCoA.^59,60^ Permutational Analysis of Variance (PERMANOVA) was used to estimate associations in beta diversity between groups through the vegan package.^61^ ANCOMBC was used to determine differentially abundant ASVs between groups at the genus level whenever possible. The Benjamini-Hochberg correction for multiple comparisons applied to all p-values. A p-value of ≤0.05 was used for all statistical tests.

## Supplementary Material

Supplemental Material

Supplemental Material

## Data Availability

Data openly available at SRA at NCBI under the accession number PRJNA1039832. Link will be available on release date of: 2024-05-01 at https://www.ncbi.nlm.nih.gov/sra/PRJNA1039832
