# Supplementary figures and images for: Vaginal and rectal microbiome changes following administration of a multi-species antenatal probiotic: A randomized control trial
Source: Gut Microbes Rep. 2024 Apr 19;1(1):2334311. doi: 10.1080/29933935.2024.2334311 (PMC11065196; doi:10.1080/29933935.2024.2334311)

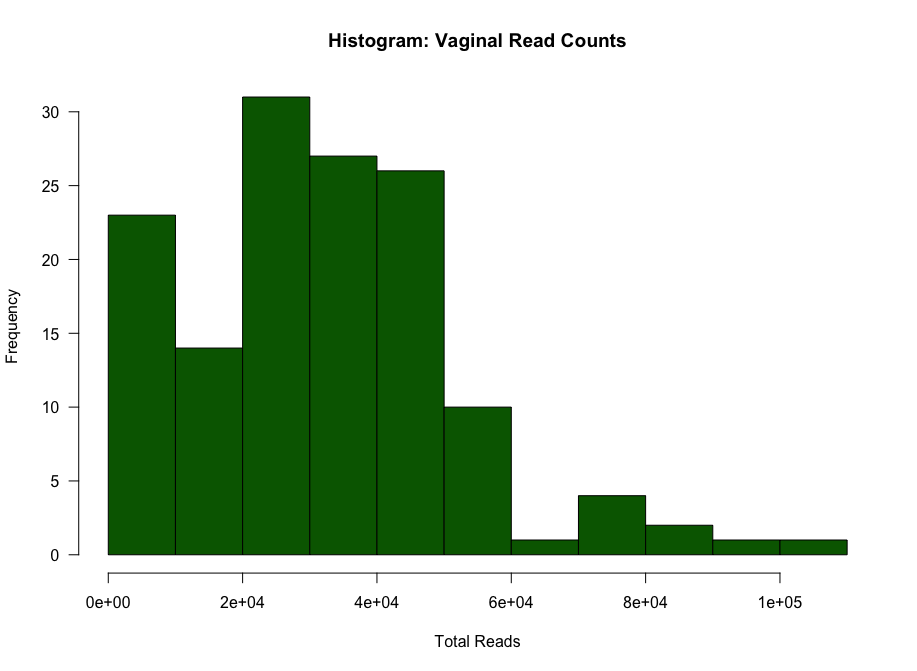

Supplement: Supplemental Material [file KGMR_A_2334311_SM6415.tiff]

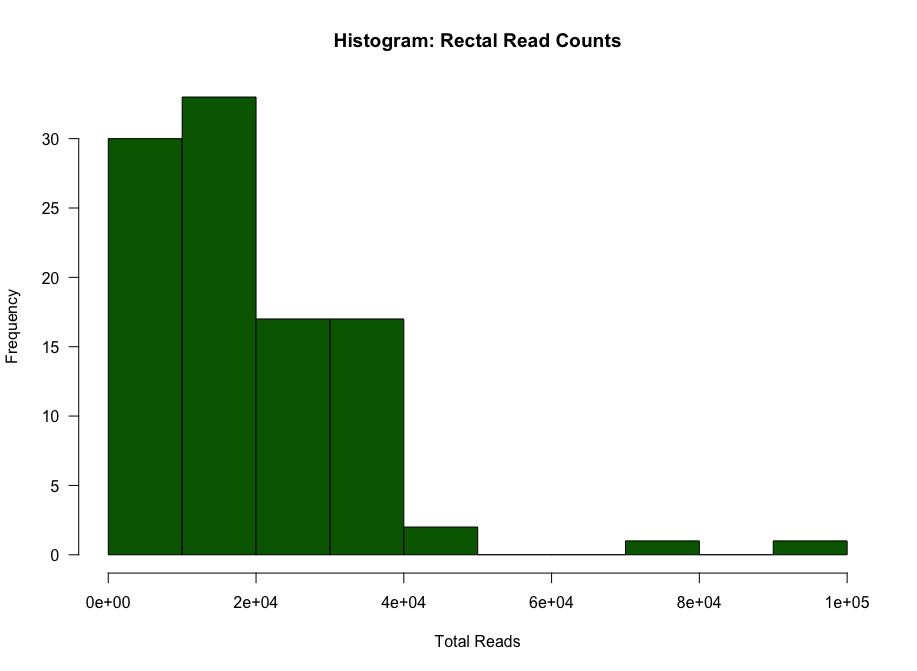

Supplement: Supplemental Material [file KGMR_A_2334311_SM6407.tiff]
